# Synthesis, characterization and assessment of anti-quorum sensing activity of copper(II)-ciprofloxacin complex against *Pseudomonas aeruginosa* PAO1

**DOI:** 10.1186/s13568-020-01017-3

**Published:** 2020-04-24

**Authors:** Farzaneh Rafiee, Fakhri Haghi, Rahman Bikas, Azam Heidari, Mina Gholami, Anna Kozakiewicz, Habib Zeighami

**Affiliations:** 1 Department of Microbiology and Virology, School of Medicine, Zanjan University of Medical Sciences, Zanjan, Iran; 2 Department of Chemistry, Faculty of Science, Imam Khomeini International University, Qazvin, 34148-96818 Iran; 3 School of Medicine, Zanjan University of Medical Sciences, Zanjan, Iran; 4 Department of Biomedical and Polymer Chemistry, Faculty of Chemistry, Nicolaus Copernicus University in Torun, 87-100 Toruń, Poland

**Keywords:** Ciprofloxacin, Metal complex, *Pseudomonas aeruginosa*, Quorum sensing

## Abstract

Quorum sensing (QS) inhibition by metal-antibiotic complexes is a promising strategy for the management and control of multidrug resistant *Pseudomonas aeruginosa* infections. We investigated the anti-quorum sensing activity of sub-minimum inhibitory concentration (sub-MIC) of copper(II) sulfate pentahydrate-ciprofloxacin (Cu-CIP) complex and free ciprofloxacin (free-CIP) against *P. aeruginosa* PAO1. Copper-CIP complex was synthesized and its characterization was assessed using spectroscopic methods and single crystal X-ray analysis. The effect of sub-MIC (1/4 and 1/16 MIC) concentrations of Cu-CIP and free-CIP on cell growth, biofilm formation, motility, alginate and pyocyanin production, H_2_O_2_ susceptibility and expression of QS circuit genes *lasI* and *lasR* in PAO1 was determined. Minimum inhibitory concentration of Cu-CIP complex and free-CIP was determined as 0.125 µg/ml. Copper-CIP complex did not show significant effect on the cell growth at concentrations of 1/4 and 1/16 MIC. However, sub-MIC concentrations (1/4 and 1/16 MIC) of Cu-CIP showed the significant reduction in violacein production, motility, biofilm formation, alginate and pyocyanin production and sensitivity to H_2_O_2_ in a concentration dependent manner (P < 0.001). Copper-CIP at the concentration of 1/4 MIC showed the greatest reduction in *lasI* and *lasR* transcriptional expression (89.5% and 96.2% respectively). Considering the biological effects of Cu-CIP complex and its inhibitory activity on QS related virulence traits at low concentrations (0.03 and 0.007 µg/ml), it may be used as an effective approach in the management of infections caused by *P. aeruginosa.*

## Key points

- Our results indicate the potential of Cu-CIP complex at the concentration of 1/4 MIC to inhibit biofilm formation and QS related genes and virulence traits.
- Considering the beneficial biological effects of this metal complex and its inhibitory effects on biofilm formation it may be used as an effective approach in the management of infections caused by *P. aeruginosa*.

## Introduction

*Pseudomonas aeruginosa* as an important nosocomial pathogen can causes serious infections such as cystic fibrosis, endocarditis, bacteremia, wound, burn and urinary tract infections. The pathogenicity of *P. aeruginosa* is related to different virulence factors like biofilm formation and the production of exotoxins, elastase, alginate and sidrophores (Klrissa and Katouli 2016). Resistance to antimicrobial agents and biofilm formation have made it as a serious problem in hospitalized patients (Amini and Namvar 2019). Conventional antimicrobial agents are usually inefficient in the treatment of chronic and persistent infections of *P. aeruginosa*. Development of new therapeutic approaches is necessary to prevent infections caused by drug resistant *P. aeruginosa* (Starkey et al. 2014). Inhibition of quorum sensing (QS) is an effective approach for development of anti-pathogenic agents and control of microbial infections. Quorum sensing, an intercellular communication system, is involved in control of gene expression in response to cell density (Starkey et al. 2014). *Pseudomonas aeruginosa* has three distinct QS systems including LasI/LasR, RhlI/RhlR, and PQS/MvfR which utilize small diffusible signal molecules of the N-acyl homoserine lactones (AHL) family called autoinducers (AIs). The LasI/R and RhlI/R systems regulate the expression of virulence factors like exotoxin A, LasA protease, LasB elastase, Apr alkaline protease, rhamnolipids, pyocyanin and hydrogen cyanide through signal molecules (Curutiu et al. 2018; Hentzer et al. 2003; Lee and Zhang 2015). Therefore, inhibitors of quorum sensing can be used to attenuate the virulence and pathogenesis and may have a role in control and treatment of acute and persistent infections (Azam et al. 2012; Sadeek et al. 2015). Previous studies showed that the subinhibitory concentrations (sub-MIC) of antibiotics may affect virulence factors like cell surface hydrophobicity, attachment and biofilm formation, motility and sensitivity to oxidative stress. Ciprofloxacin is a broad spectrum antimicrobial agent that has been extensively used for the treatment of several bacterial infections caused by Gram negative and Gram positive strains (Sabale et al. 2012; Uivarosi 2013). Subinhibitory concentrations of ciprofloxacin significantly decreased the virulence factors like motility, biofilm formation, and production of protease, elastase, siderophore, alginate, and rhamnolipid in *P. aeruginosa* (Khan et al. 2017; Gupta et al. 2016). According to previous studies, the metal-antibiotic complexes possess more biological and antimicrobial activity than free ligands (Sadeek et al. 2015; Sabale et al. 2012). Metal complexes are promising agents for improvement of antimicrobial activity. This property may be explained by chelation theory and overtone concept. According to this theory, the overlapping of the ligand orbital and the partial sharing of the donor groups with positive charge of the metal ion lead to reduction of the metal ion polarity. Furthermore, it enhances delocalization of pi-electrons over the whole chelate ring and increases the lipophilicity and penetration of the metal complex into the membrane lipids and blokes the binding sites of metal dependent proteins of the microorganism (Sadeek et al. 2015; Sabale et al. 2012; Uivarosi 2013; Khan et al. 2017). Previous studies have been performed on the antibacterial activities of some metal-ciprofloxacin complexes but the effect of these compounds on *P. aeruginosa* QS system has not been demonstrated. So, this study aimed to investigated the anti-quorum sensing activity of sub-MIC concentrations of copper(II) sulfate pentahydrate (CuSO_4_·5H_2_O)-ciprofloxacin complex (Cu-CIP) and free ciprofloxacin (free-CIP) against *P. aeruginosa* PAO1.

## Materials and methods

### Materials and instrumentation

Copper(II) sulfate pentahydrate (CuSO_4_·5H_2_O) and ciprofloxacin were purchased from Sigma-Aldrich (Buchs, Switzerland) and solvents were bought from Merck (Germany). The elemental analyses (carbon, hydrogen and nitrogen) of the complex were obtained from a Carlo ERBA Model EA 1108 analyzer. The content of copper in the complex was determined by atomic absorption analysis on a Varian Spectra AA-220 equipment. Fourier transform infrared (FT-IR) spectroscopy was performed using a FT-IR Spectrometer Bruker Tensor 27 as KBr disks. Fresh stock solutions of Cu-CIP and free-CIP were prepared in distilled water at the concentration of 1 mg/ml. *N*-Acyl-homoserine lactone (C6-HSL) (Sigma-Aldrich) was used at 20 µM.

### Synthesis of Cu(II)-ciprofloxacin complex

The Cu-CIP complex was synthesized using the reaction of ciprofloxacin (2.0 mmol, 0.663 g) and CuSO_4_·5H_2_O (2.0 mmol, 0.499 g) in ethanol (20 ml) and the crystals were directly obtained by thermal gradient method. Briefly, ciprofloxacin and CuSO_4_·5H_2_O were added to a branched tube and the tube was filled with ethanol. Then, the arm was immersed in an oil bath at 60 °C. After 3 days, cubic green crystals of Cu-CIP were formed which filtered off, washed with cold ethanol and dried at air. Yield: 87% (1.06 g). *Anal*. Calc. for C_19_H_32_CuFN_3_O_12_S (MW = 609.08 g/mol): C, 37.47; H, 5.30; N, 6.90; Cu, 10.43%. Found: C, 37.50; H, 5.28; N, 6.86; Cu, 10.46%. FT-IR (KBr, cm^−1^): 3515 (br, m); 3507 (br, m); 3246 (br, m); 3032 (br, m); 2991 (br, w); 2860 (br, w); 2757 (br, w); 2536 (w); 1632 (s); 1629 (s); 1580 (s); 1553 (m); 1525 (m); 1495 (m); 1455 (m); 1420 (m); 1381(m); 1358 (m); 1340 (m); 1307 (m); 1295 (m); 1269(m); 1254 (m); 1197 (m); 1183 (m); 1171 (m); 1150 (m); 1134 (m); 1131 (m); 1119 (m); 1103 (m); 1100 (m); 1057 (m); 1036 (m); 1022 (m); 973 (w); 950 (m); 926 (w); 856 (w); 815 (w); 757 (w); 709 (w); 629 (w); 617 (w); 539 (w); 508 (w); 498 (m); 463 (m). The Cu-CIP complex was obtained as quite pure single crystals and the X-ray structure was determined.

### Single crystal X-ray analysis

Crystals of the Cu-CIP complex suitable for the X-ray diffraction experiment were obtained from the ethanol. The diffraction data were collected at room temperature using Oxford Sapphire CCD diffractometer, MoKα radiation λ = 0.71073 Å, by ω-2θ method. The Cu-CIP structure was solved by direct methods and refined with the full-matrix least-squares procedure on *F*^*2*^ (SHELX-2014 program packages (Sheldrick 2015). The analytical absorption correction was applied for the studied crystal, based on the crystal shape (Software 2015). All heavy atoms were refined with anisotropic displacement parameters. Positions of H atoms have been found from the electron density maps and hydrogen atoms were constrained in the refinement with the appropriate riding model as implemented in SHELX during refinement. The results of the data collection and refinement are summarized in Table 1. The structure Cu-CIP has been deposited at the Cambridge Crystallographic Data Centre, the deposition number is 1947979.

**Table 1:** Crystallographic data and refinement details for Cu-CIP

Compound	Cu-CIP
Formula	C_17_H_32_CuFN_3_O_12_S
*M*_r_/(g/mol)	609.08
Crystal shape, color	Cubic, green
Crystal size/mm^3^	0.30 × 0.24 × 0.13
λ/Å	0.71073 (Mo *Kα*)
Crystal system	Monoclinic
Space group	*P2*_*1*_*/c*
*a*/Å	15.9488 (12)
*b*/Å	11.0141(9)
*c*/Å	14.6494 (12)
*β*/°	106.958 (9)
*V*/Å^3^	2461.4 (4)
*Z*	4
*D*_calc_/(g/cm^3^)	1.644
*μ*/mm^−1^	1.05
*F*(000)	1268
*θ* range/°	2.3–28.6
*h,k,l*	− 20 → 20 − 14 → 14 − 19 → 18
Measured reflections	16,651
Independent reflections	5682
Reflections with *I* > 2*σ*(*I*)	4365
*R*_int_	0.038
*R*[*F*^*2*^> 2σ*(F*^*2*^)]	0.041
*wR(F*^*2*^*)*	0.126
*S*	1.06
Abs. correction	Analytical
Parameters	334
Restraints	0
Δρ_max_/Δρ_min_	0.74 −0.65

### Bacterial strains and growth media

*Pseudomonas aeruginosa* PAO1 was used for the assay of anti-quorum sensing activity of Cu-CIP complex and free-CIP. *Chromobacterium violaceum* CV026 was used as reporter strain. The cultures were grown in Luria–Bertani (LB) broth/agar (Merck, Germany) aerobically at 37 °C or 30 °C for 16–18 h. Bacteria were preserved in Tryptic Soy Broth (TSB, Merck, Germany) containing 10% (v/v) glycerol at − 80 °C.

### Minimum inhibitory concentration

Minimum inhibitory concentration (MIC) of Cu-CIP and free-CIP was determined using the broth microdilution method (Aziz Zahid et al. 2017). Twofold serial dilutions of compounds (16, 8, 4, 2, 1, 0.5, 0.25, 0.125, 0.06, 0.03 µg/ml) were prepared and inoculated with 5 × 10^6^ CFU/ml of PAO1 overnight culture. After 24 h of incubation at 37 °C, MIC was calculated as the lowest concentration of compound that inhibited visible growth of PAO1.

### Cell growth analysis

The growth of both untreated and treated PAO1 was measured by broth microdilution method (Aziz Zahid et al. 2017). Luria–Bertani broth containing the different concentrations of Cu-CIP or free-CIP (0.003 to 0.5 µg/ml) was inoculated with PAO1 and incubated at 37 °C for 16 h. The absorbance was measured at OD_630_ nm and the percentage of cell growth was calculated as follow: OD_630_ (treated PAO1)/OD_630_ (untreated PAO1) × 100.

### Biosensor bioassay

Anti-quorum sensing activity of Cu-CIP and free-CIP was assessed by *C. violaceum* CV026 strain as described previously (Heidari et al. 2017). Fifty microliters of compounds at the concentrations of 1/4 and 1/16 MIC were loaded onto wells of 5 mm on the surface of LB agar inoculated with *C. violaceum* CV026 and supplemented with 20 µM of C6-HSL. Quorum sensing inhibition was determined after 24 h of incubation at 30 °C by the presence of a zone of pigmentless but viable cells around the well. Dimethyl sulfoxide (DMSO) was used as a control.

### Effects on biofilm formation

The microtiter plate assay was carried out to determine the effect of Cu-CIP and free-CIP on the biofilm formation of PAO1 (Roudashti et al. 2017). Treated (concentration of 1/4 and 1/16 MIC of Cu-CIP or free-CIP) and untreated cultures of PAO1 in LB broth were incubated overnight at 37 °C. Free cells were removed and biofilms were washed three times with sterile PBS and fixed with 99% (v/v) methanol. The adhered cells were stained with 0.2% (w/v) crystal violet (HiMedia, India). The additional crystal violet was removed by washing with distilled water. Finally, crystal violet was solubilized using 33% (v/v) glacial acetic acid for 20 min and the absorbance was measured at OD_590_ nm. The mean of three measurements was reported. The percent inhibition of biofilm formation was calculated as follow: (1 − OD_590_ of treated PAO1/OD_590_ of untreated PAO1) × 100.

### Motility assays

Treated (concentrations of 1/4 and 1/16 MIC of Cu-CIP or free-CIP) and untreated agar plates were used to assess the swarming and twitching motilities (Bahari et al. 2017).

*Swarming* Nutrient agar (0.5% Bacto agar) supplemented with 5% glucose with and without sub-MIC concentrations (1/4 and 1/16 MIC) of compounds was prepared and inoculated with 2 µl of PAO1 culture and incubated at 37 °C for 16 h.

*Twitching* Treated and untreated LB agar (1%) plates were prepared and PAO1 cultures were stabbed with a sterile toothpick through the agar layer to the bottom of plate. After 48 h of incubation at 37 °C, the agar layer was removed and cells attached to plate were stained with crystal violet (0.1%, w/v). Then, the stained zone diameter was measured.

### Effects on production of alginate

The effect of Cu-CIP and free-CIP on the alginate production of PAO1 was assayed according to previous study (Hoffmann et al. 2007). Briefly, 5 ml of LB broth with and without sub-MIC concentrations (1/4 and 1/16 MIC) of compounds were inoculated with 500 µl of PAO1 suspension (1 × 10^8^ CFU/ml) and incubated at 37 °C. After 24 h of incubation, 1 ml of culture was centrifuged at 12,000 rpm for 30 min and the supernatant was maintained at 80 °C for 30 min. The supernatant was then centrifuged at 12,000 rpm for 30 min and precipitated with ice-chilled ethanol 99% (v/v) at 4 °C for 2 h and mixed with 1 ml of sterile saline (0.9%). Finally, 1 ml of borate sulfuric acid reagent (100 mM H_3_BO_3_ in concentrated H_2_SO_4_) and 34 µl of carbazole reagent (0.1% in ethanol) were added to 118 µl of sample on ice. This mixture was heated to 55 °C for 30 min and the absorbance was measured at OD_530_ nm.

### Effects on pyocyanin production

Production of pyocyanin was assessed in a quantitative chemical assay (Hoffmann et al. 2007). Briefly, 10 ml of treated (concentrations of 1/4 and 1/16 MIC of Cu-CIP or free-CIP) and untreated LB broth were inoculated with 500 µl of PAO1 suspension (1 × 10^8^ CFU/ml) and incubated at 37 °C for 24 h. Five ml of cell free supernatant was mixed with 3 ml of chloroform. The phase extracted with chloroform was transferred to another test tube and 1 ml of HCl (0.2 M in distilled water) was added. After centrifugation at 12,000 rpm for 10 min, the absorbance of red phase was measured at OD_520_ nm. The pyocyanin concentration was determined as µg/ml = (OD_520_ × 17.072).

### Effects on hydrogen peroxide susceptibility

Hydrogen peroxide susceptibility of untreated and treated PAO1 cultures was determined according to previous studies (He et al. 2014; Driscoll et al. 2007). Briefly, 2 ml of treated (concentrations of 1/4 and 1/16 MIC of Cu-CIP or free-CIP) and untreated LB broth were inoculated with 100 µl of PAO1 suspension (1 × 10^8^ CFU/ml) and incubated at 37 °C for 24 h. After centrifugation at 5000 rpm for 10 min, 100 µl of the supernatant were poured onto LB agar plates and a filter paper saturated with H_2_O_2_ (10%, v/v) was placed in the center of plates. After 24 h of incubation at 37 °C, the inhibition zone diameter was measured and reported in mm.

### Extraction of RNA and cDNA synthesis

RNA was extracted at the middle of the exponential growth phase of treated and untreated PAO1 cultures by EZ-10 Spin Column Total RNA Miniprep Super Kit (Bio Basic, Canada) with on-column DNaseI digestion (Bio Basic, Canada) according to the kit handbook. The purity and concentration of extracted RNA samples were determined by NanoDrop Spectrophotometer (ND-1000, Nano-Drop Technologies, Wilmington, DE). Complementary DNA (cDNA) was then synthesized using PrimeScript™ RT reagent Kit (Takara, Japan) in a total volume of 10 µl containing 0.5 µl of PrimeScript RT Enzyme Mix I, 2 µl of 5× PrimeScript Buffer, 0.5 µl of Random 6mers (100 μM), 500 ng of RNA and RNase free dH_2_O. The reactions were incubated at 37 °C for 30 min, 85 °C for 5 s and 4 °C for 10 min.

### Real time PCR

The effect of sub-MIC concentrations (1/4 and 1/16 MIC) of Cu-CIP and free-CIP on expression of *lasI* and *lasR* genes of PAO1 was assessed as described previously (Bahari et al. 2017; Roudashti et al. 2017). Real time PCR reaction mixture was composed of 10 µl of TB Green Premix Ex Taq (Takara, Japan), 0.4 µl of ROX Reference Dye (50×), 0.4 µl of each primer (10 µM), 1 µl of cDNA (100 ng) and 7.8 µl of sterile purified water to complete the volume. Assays were carried out with an Applied Biosystems StepOnePlus™ Real-Time PCR System in triplicate. The expression level of *lasI* and *lasR* genes was normalized to the expression of reference gene *oprL*. Analysis of melting curve showed that the accumulation of TB Green-bound DNA was specific for *lasI* or *lasR* genes. The no template control (NTC) and no reverse transcriptase control (no-RT) were included in all experiments. The expression of treated cultures was compared with untreated cultures and the data were analysed by 2^−ΔΔCt^ method (Cuprys et al. 2018).

### Statistical analysis

All data were analyzed using SPSS version 17.0 software. The statistical analysis of data was carried out using one-way analysis of variance (ANOVA). Differences with *P* value < 0.05 were considered significant.

## Results

### Synthesis and characterization of Cu-CIP complex

The reaction of ciprofloxacin with copper(II) sulfate pentahydrate in ethanol gave green crystals by thermal gradient method. In the FT-IR spectrum of the crystals, the peaks at 1629, 1580, 1254 and 3032 cm^−1^ can be attributed to the C=N, C=C, C–O, and C–H bond vibrations. The peak at 1632 cm^−1^ is due to the coordinated carboxylate (–CO_2_) group which confirms the coordination of carboxylate group to the copper(II) ion. The band at 1171 cm^−1^ is due to the C-F vibration and the broad bands at about 3500 and 3246 cm^−1^ are due to the OH and NH groups, respectively which are involved in intermolecular hydrogen bond interactions.

**Fig. 1:**
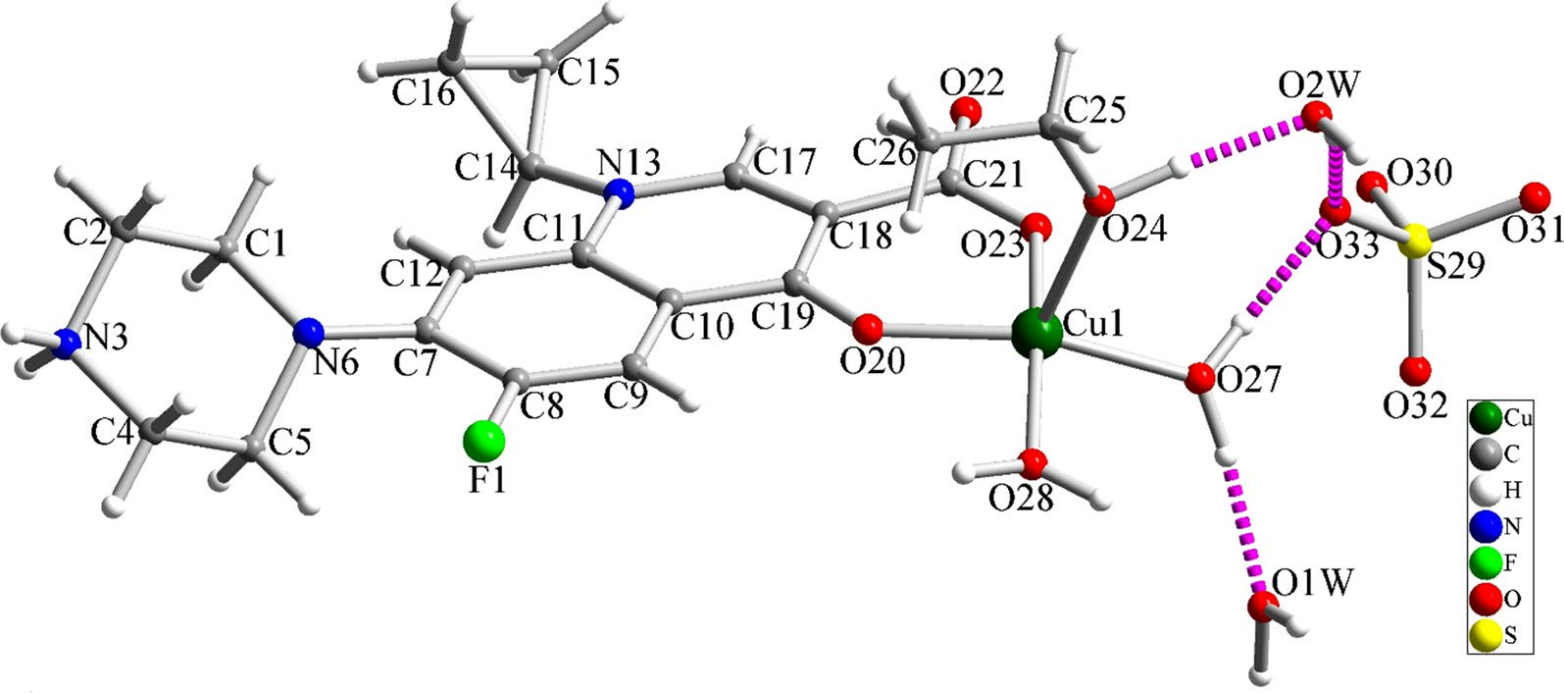
The molecular structure of Cu-CIP with atom labeling. Pink dashed lines show hydrogen bond interactions

**Fig. 2:**
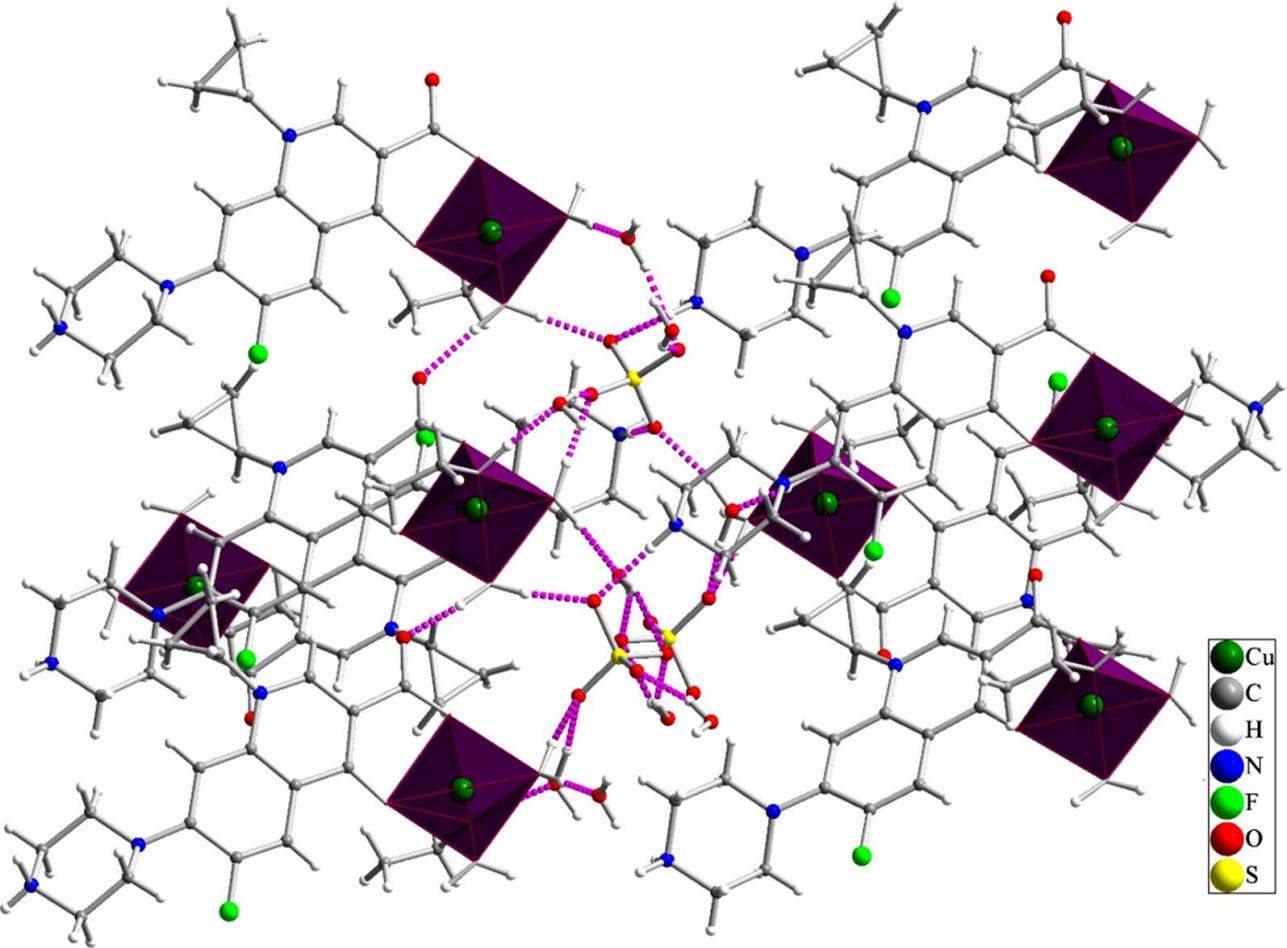
Intermolecular hydrogen bond interactions (pink dashed lines) in the crystal structure of Cu-CIP

The structure of green crystals was characterized using single crystal X-ray analysis. Copper-CIP molecular structure is shown in Fig. 1 and selected bond lengths and angles are presented in Table 2. Diffraction studies indicated that Cu-CIP is crystallized in *P2*_*1*_*/c* space group of monoclinic system and is a mononuclear complex of Cu(II). The complex is cationic and a sulfate anion is located beside it. The coordination environment around Cu(II) ion is generated by coordination of two oxygen atoms (carboxylate and ketonic groups) from ciprofloxacin, two oxygen atoms from coordinated water molecules and one oxygen atom from coordinated ethanol solvent. The coordination environment can be described as square pyramidal geometry with the τ value of 0.0945 (τ = difference between the two largest angles/60; τ = 1 for ideal trigonal–bipyramidal and τ = 0 for ideal square-pyramidal). During the formation of Cu-CIP, the hydrogen atom of carboxylic acid functionality is eliminated and the nitrogen atom of piperazine ring is protonated. Therefore, ciprofloxacin acts as a neutral bidentate O_2_-donor ligand in Cu-CIP. By considering the presence of Cu(II), the complex molecule is a cationic complex and the + 2 positive charge is balanced by − 2 sulfate anion. There are three uncoordinated water molecules in the crystal structure of Cu-CIP which are stabilized in the crystal by strong and directed hydrogen bond interactions. The sulfate anion is also stabilized in the crystal structure of Cu-CIP by involving in hydrogen bond interactions. Each oxygen atoms of the SO_4_^2−^ anion involves two hydrogen bond interactions which have considerable effect in stabilization of the crystal structure. A part of the hydrogen bond interactions in the crystal structure of Cu-CIP is shown in Fig. 2 and the detailed information of hydrogen bond interactions is presented in Table 2. These intermolecular interactions connect the molecules to each other and create 3D polymeric network (see Figs. 1 and 2).

**Table 2:** Selected bond lengths (Å) and bond angles (°) in the crystal structure of Cu-CIP

Bond	Length/Å	Bond	Angle/°
Cu1–O20	1.9121(18)	O20–Cu1–O23	92.86(8)
Cu1–O23	1.917(2)	O20–Cu1–O27	164.11(10)
Cu1–O27	1.956(2)	O23–Cu1–O27	88.95(9)
Cu1–O28	1.969(2)	O20–Cu1–O28	86.14(8)
Cu1–O24	2.256(3)	O23–Cu1–O28	169.78(10)
C8–F1	1.354(3)	O27–Cu1–O28	89.28(9)
C2–N3	1.492(3)	O20–Cu1–O24	103.45(10)
N3–C4	1.482(3)	O23–Cu1–O24	97.15(11)
N13–C17	1.343(3)	O27–Cu1–O24	91.97(10)
N13–C14	1.461(3)	O28–Cu1–O24	92.97(10)
C11–N13	1.401(3)

### MIC determination

Copper-CIP and free-CIP MIC against PAO1 was determined as 0.125 µg/ml. Sub-inhibitory concentrations of Cu-CIP and free-CIP corresponding to 1/4 and 1/16 MIC (0.031 and 0.007 µg/ml, respectively) were applied to assess QSI activity.

### Cell growth analysis

**Fig. 3:**
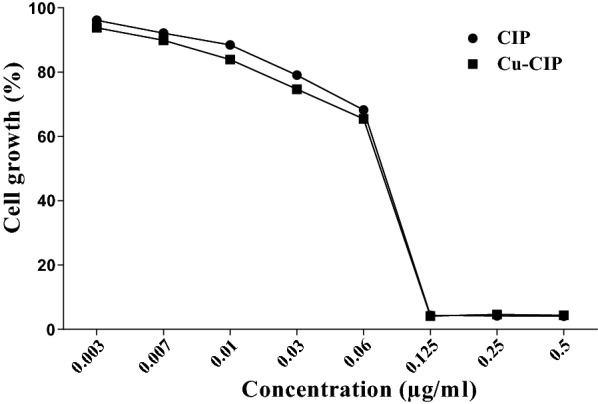
The effect of concentrations of 0.003 to 0.5 µg/ml of ciprofloxacin (CIP) and copper-ciprofloxacin complex (Cu-CIP) on cell growth of PAO1

The effect of different concentrations of Cu-CIP and free-CIP on cell growth of PAO1 is shown in Fig. 3. The concentration of 0.125 µg/ml of Cu-CIP or free-CIP (1 × MIC) significantly decreased the cell growth in comparison with untreated PAO1. However, the cell growth was not significantly reduced at the concentrations of 1/4 and 1/16 MIC of compounds compared with untreated control.

### Biosensor bioassay

Copper-CIP and free-CIP showed anti-quorum sensing activity in *C. violaceum* CV026 biosensor bioassay. The concentrations of 1/4 and 1/16 MIC of free-CIP showed the pigmentless zones of 16 and 11 mm, respectively, indicating the violacein inhibition around the wells. Whereas, Cu-CIP showed stronger anti-quorum sensing activity at the concentrations of 1/4 and 1/16 MIC with pigmentless zones of 23 and 18 mm, respectively.

### Effects on biofilm formation

**Fig. 4:**
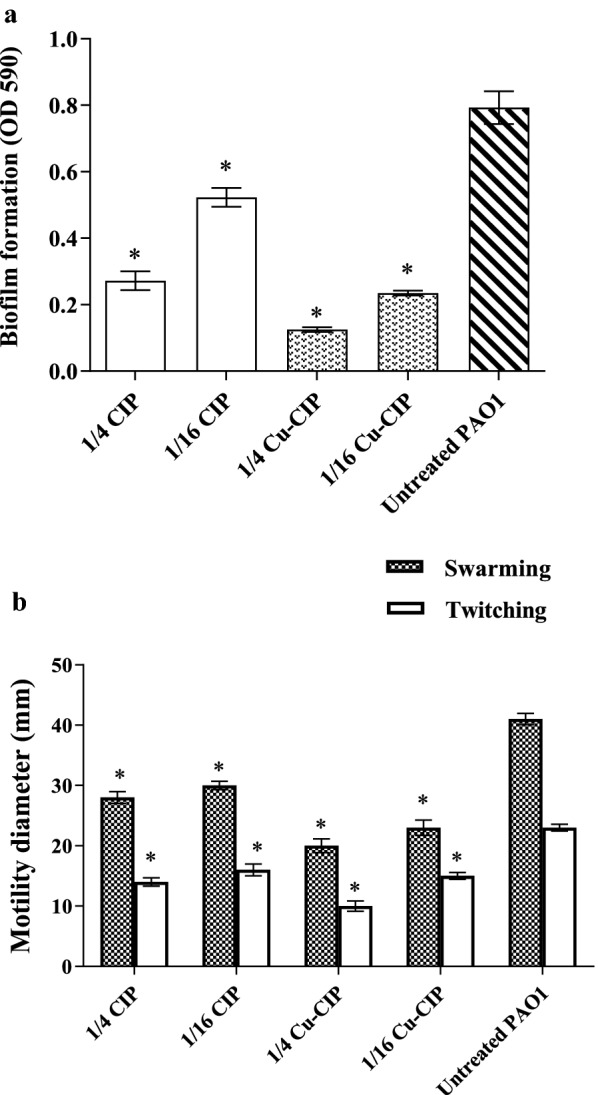
Effect of subinhibitory concentrations (1/4 and 1/16 MIC) of ciprofloxacin (CIP) and copper-ciprofloxacin complex (Cu-CIP) on **a** biofilm formation and **b** swarming ■ and twitching □ motilities (*, significant, P < 0.05)

The biofilm formation was inhibited in the presence of Cu-CIP or free-CIP between 34 and 84% (Fig. 4a). The inhibitory effect was concentration dependent. Biofilm inhibition was significantly higher at the concentrations of 1/4 and 1/16 MIC of Cu-CIP compared with free-CIP (*P *< 0.001). Furthermore, the concentration of 1/4 MIC of Cu-CIP demonstrated the most inhibitory effect on biofilm formation with 84% reduction (*P *< 0.001).

### Effects on motility

Swarming and twitching motilities were significantly decreased at the concentrations of 1/4 MIC and 1/16 MIC of Cu-CIP or free-CIP in comparison with untreated PAO1 (*P *< 0.05) (Fig. 4b). There was a 56.5% decrease in twitching and 51.2% decrease in swarming motility at the concentration of 1/4 MIC of Cu-CIP. The rate of motility reduction was significantly higher in the presence of Cu-CIP compared with free-CIP (*P *< 0.05).

### Effects on alginate production

**Fig. 5:**
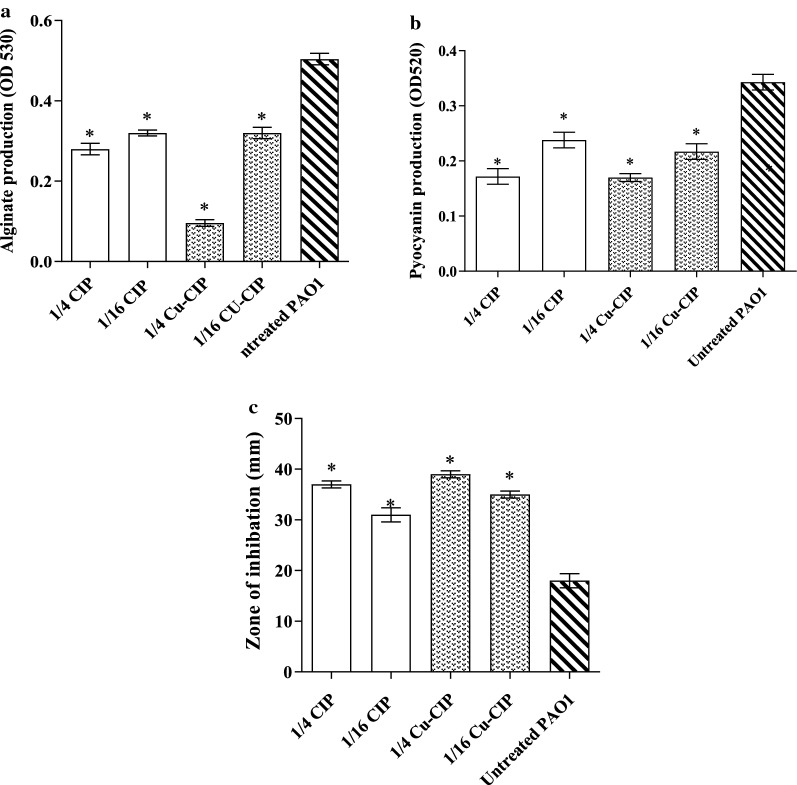
Effect of subinhibitory concentrations (1/4 and 1/16 MIC) of ciprofloxacin (CIP) and copper-ciprofloxacin complex (Cu-CIP) on **a** the alginate production, **b** pyocyanin production and **c** H_2_O_2_ susceptibility (*, significant, P < 0.05)

As shown in Fig. 5a, the concentrations of 1/4 and 1/16 MIC of Cu-CIP or free-CIP exhibited 36.5% to 80.9% reduction in alginate production compared with untreated PAO1 (P < 0.05). The most inhibitory effect on alginate production was detected at the concentration of 1/4 MIC of Cu-CIP with 80.9% reduction (*P *< 0.001).

### Effects on pyocyanin production

The effect of Cu-CIP and free-CIP on pyocyanin production is shown in Fig. 5b. Pyocyanin production was significantly reduced (ranging from 21 to 50%) at the concentrations of 1/4 and 1/16 MIC of Cu-CIP or free-CIP in comparison with untreated PAO1 (*P *< 0.05). There was no significant difference in pyocyanin reduction between Cu-CIP and free-CIP at the concentrations of 1/4 and 1/16 MIC.

### Effects on hydrogen peroxide susceptibility

The concentrations of 1/4 and 1/16 MIC of Cu-CIP or free-CIP significantly increased the sensitivity of PAO1 to H_2_O_2_ in comparison with untreated PAO1 (ranged from 1.7- to 2.1-fold) (*P *< 0.05) (Fig. 5c). The concentration of 1/4 MIC of Cu-CIP showed the most sensitivity to H_2_O_2_ by 2.1 fold (*P *< 0.05).

### Expression of *lasI* and *lasR*

**Fig. 6:**
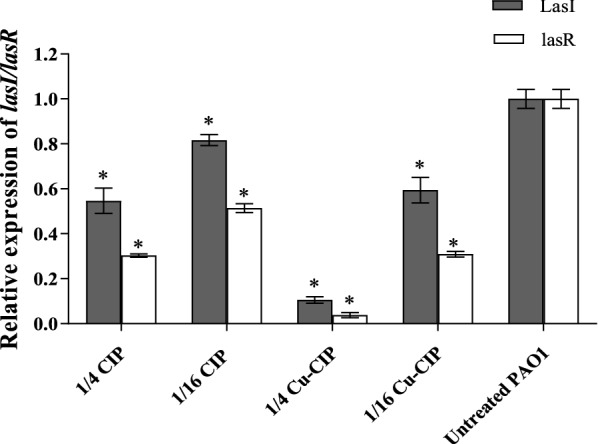
The subinhibitory concentrations (1/4 and 1/16 MIC) of ciprofloxacin (CIP) and copper-ciprofloxacin complex (Cu-CIP) inhibited QS regulated genes in treated PAO1 (*, significant, P < 0.05)

Relative expression of *lasI* and *lasR* genes was determined from Ct values and standard curves. The melting curve analysis showed the same profiles without dimer primer formation. The standard curve of *oprL* and target genes of *lasI* and *lasR* showed R2 values 0.99–0.97. Relative expression of treated cultures was compared with untreated cultures and the data were analyzed using 2^−ΔΔCt^ method. Copper-CIP and free-CIP significantly repressed the expression of *lasI* and *lasR* between 18.3 and 96.2% relative to untreated PAO1 (*P *< 0.05) (Fig. 6). The concentration of 1/4 MIC of Cu-CIP showed the greatest reduction in *lasI* and *lasR* expression (89.5% and 96.2% respectively) (*P *< 0.001). The reduction in *lasI* and *lasR* expression was significantly higher in the presence of Cu-CIP compared with free-CIP (*P *< 0.05).

## Discussion

Inhibition of quorum sensing by metal-drug complexes is a promising strategy for control of drug resistant bacterial infections (Driscoll et al. 2007; Sabale et al. 2012; Uivarosi 2013; Khan et al. 2017). Previous studies have been reported the anti-tumor, anti-fungal, anti-viral, anti-bacterial and special biological activities of metal complexes (Sabale et al. 2012; Khan et al. 2017). It has been found that the metal complexes of quinolones possess more biological and antibacterial activities than free quinolones (Sabale et al. 2012). However, the effect of metal complexes of ciprofloxacin on *P. aeruginosa* QS system has not been demonstrated. As far as we know, this is the first study to demonstrate anti-quorum sensing activity of sub-MIC concentrations of Cu-CIP complex against *P. aeruginosa*. According to our results, minimum inhibitory concentration of Cu-CIP complex and free-CIP was determined as 0.125 µg/ml. Cuprys et al. (2018) also reported similar results on MIC values of Cu-CIP complex and free-CIP against *P. aeruginosa* (Cuprys et al. 2018). Copper-CIP and free-CIP decreased the cell growth of PAO1 at concentrations of 1 × MIC. However, the cell growth was not significantly reduced at concentrations of 1/4 and 1/16 MIC, proving that their anti-quorum sensing activities were obtained by QS inhibition not through bacterial cell killing. Similar results were reported by El-Mowafy et al. (2014) which showed that sub-MIC concentrations of aspirin significantly decreased the quorum sensing signals of *P. aeruginosa* without reduction in cell growth (El-Mowafy et al. 2014). In the lack of significant effect of Cu-CIP on the cell growth at concentrations of 1/4 and 1/16 MIC, it demonstrated anti-quorum sensing activity in *C. violaceum* CV026. Free-CIP also showed a concentration-dependent reduction in violacein production. However, Cu-CIP complex showed stronger anti-quorum sensing activity in comparison with free-CIP. Similar to our results, sub-MIC concentrations of compounds such as curcumin, furanone and its derivatives, pyridoxal lactohydrazone, cinnamaldehyde and its derivatives, iberin, ajoene, catachin and some antibiotics significantly inhibited QS signaling (Heidari et al. 2017; Roudashti et al. 2017; Bahari et al. 2017; El-Mowafy et al. 2014; Tang and Zhang 2014; Manner and Fallarero 2018).

Biofilm formation is an important virulence factor involved in the pathogenicity, drug resistance and development of chronic and persistent infections of *P. aeruginosa* (Amini and Namvar 2019; Lee and Zhang 2015). Since quorum sensing system is an important regulating factor in the biofilm formation, its inhibition may promote the eradication of biofilms (Hentzer et al. 2003). The concentration of 1/4 MIC of Cu-CIP complex demonstrated the most inhibitory effect on biofilm formation with 84% reduction. Furthermore, biofilm inhibition was significantly higher in the presence of Cu-CIP compared with free-CIP. In study conducted by Tewes et al. (2019), efficacy of ciprofloxacin and its copper complex was determined against *P. aeruginosa* biofilms. According to their reports, there was no difference between free-CIP and Cu-CIP in terms of efficacy against biofilm (Tewes et al. 2019). In accordance with our results, Packiavathy et al. (2012) and Gopu et al. (2015) also reported a significant decrease in biofilm formation of food-borne pathogens treated with 10 µg/ml of methyl eugenol or 40 µg/ml of quercetin (Packiavathy et al. 2012; Gopu et al. 2015). Significant reduction in biofilm formation was also reported at sub-MIC concentrations of curcumin in combination with ciprofloxacin, ceftazidime, azithromycin and gentamicin in our previous studies (Roudashti et al. 2017; Bahari et al. 2017). In study conducted by Bortolotti et al. (2019), a quorum sensing inhibitor conjugated with ciprofloxacin (ET37) showed a decrease in biofilm formation and antibiotic tolerance of clinical strains of *P. aeruginosa* (Bortolotti et al. 2019).

Swarming and twitching motilities in PAO1 treated with Cu-CIP complex or free-CIP was significantly impaired relative to untreated control. However, the rate of motility reduction was significantly higher in the presence of Cu-CIP compared with free-CIP. These findings are consistent with previous reports which demonstrated that curcumin (Bahari et al. 2017; Roudashti et al. 2017), aspirin (El-Mowafy et al. 2014), pyridoxal lactohydrazone (Heidari et al. 2017) and green synthesized nanocomposites (Alavi and Karimi 2018) inhibit swarming and twitching motilities in *P. aeruginosa.*

In our study, significant reduction in virulence factors was detected at the concentrations of 1/4 and 1/16 MIC of Cu-CIP complex. Copper-CIP complex significantly decreased the alginate (36.5–80.9%) and pyocyanin (37–50%) production compared with untreated PAO1. Heidari et al. (2017) reported that pyridoxal lactohydrazone at the concentration of 1/4 MIC decreased the alginate production up to 64% in PAO1 compared with untreated control (Heidari et al. 2017). Pyocyanin as a secondary metabolite produced by *P. aeruginosa* generates reactive oxygen radicals and therefore kills other microbes and mammalian cell during bacterial infections. According to previous reports, pyocyanin inhibition showed the bactericidal activity and increased the sensitivity to oxygen free radicals (Heidari et al. 2017). In previous studies, sub-MIC concentrations of azithromycin, ceftazidime and ciprofloxacin repressed the QS activity of *P. aeruginosa* via restraining cellular permeability and inhibiting the release of QS signal C12-HSL in the surrounding environment (Bahari et al. 2017; Roudashti et al. 2017; El-Shaer et al. 2016). Similarly, sub-MIC concentrations of *trans*-cinnamaldehyde and salicylic acid significantly decreased virulence factors and inhibited the expression of *lasI/R* and *rhlI/R* systems in PAO1 (Ahmed et al. 2019). We also confirmed that PAO1 was more sensitive to H_2_O_2_ at concentration of 1/4 MIC of Cu-CIP complex. According to He et al. (2014) findings, combination of chito-oligosaccharide (COS) with azithromycin showed synergistic effects. Sub-MIC concentration of COS with azithromycin also inhibited the virulence factors and biofilm development in resistant and wild-type strains of *P. aeruginosa* (He et al. 2014).

Our study indicated that Cu-CIP complex or free-CIP significantly inhibited the expression of *lasI* and *lasR* relative to untreated PAO1. Copper-CIP showed the greatest reduction in the expression of *lasI* and *lasR* at the concentration of 1/4 MIC. Due to the important role of QS in regulation of virulence factors and biofilm formation, we speculated that Cu-CIP mediated inhibition of these factors is obtained by effects on QS.

A new Cu(II)-CIP complex was synthesized by the reaction of Cu(II) sulfate pentahydrate and ciprofloxacin in ethanol and the product was characterized using spectroscopic methods and single crystal X-ray analysis. The anti-quorum sensing activity of complex against *P. aeruginosa* was also determined. Our results indicate the potential of Cu-CIP complex at the concentration of 1/4 MIC to inhibit biofilm formation and QS related genes and virulence traits. Considering the beneficial biological effects of this metal complex and its inhibitory effects on biofilm formation and QS related virulence traits at low concentrations (0.03 and 0.007 µg/ml), it may be used as an effective approach in the management of infections caused by *P. aeruginosa.*

## Data Availability

It is the responsibility of each author for providing authenticated data and material and data would be made available also.
